# Contribution of environmental determinants to the risk of developing type 2 diabetes mellitus in a life-course perspective: a systematic review protocol

**DOI:** 10.1186/s13643-024-02488-2

**Published:** 2024-03-01

**Authors:** Yannick Wilfried Mengue, Pierre-Paul Audate, Jean Dubé, Alexandre Lebel

**Affiliations:** 1 Graduate School of Land Management and Regional Planning, Laval University, Quebec, Canada; 2 Quebec Heart and Lung Institute, Quebec, Canada; 3 National Institute of Public Health of Quebec, Quebec, Canada

**Keywords:** Land management, Neighbourhoods, Health prevention, Healthy lifestyles behaviours, Diabetes

## Abstract

**Background:**

Prevention policies against type 2 diabetes mellitus (T2DM) focus solely on individual healthy lifestyle behaviours, while an increasing body of research recognises the involvement of environmental determinants (ED) (cultural norms of land management and planning, local foodscape, built environment, pollution, and neighbourhood deprivation). Precise knowledge of this relationship is essential to proposing a prevention strategy integrating public health and spatial planning. Unfortunately, issues related to the consistency and synthesis of methods, and results in this field of research limit the development of preventive strategies. This systematic review aims to improve knowledge about the relationship between the risk of developing T2DM in adulthood and long-term exposure to its ED during childhood or teenage years.

**Methods:**

This protocol is presented according to the Preferred Reporting Items for Systematic Review and Meta-Analysis Protocols (PRISMA-P) tools. PubMed, Embase, CINAHL, Web of Science, EBSCO, and grey literature from the Laval University Libraries databases will be used for data collection on main concepts such as ‘type 2 diabetes mellitus’, ‘zoning’ or ‘regional, urban, or rural areas land uses’, ‘local food landscape’, ‘built environment’, ‘pollution’, and ‘deprivation’. The Covidence application will store the collected data for selection and extraction based on the Population Exposure Comparator Outcome and Study design approach (PECOS). Studies published until December 31, 2023, in English or French, used quantitative data about individuals aged 18 and over that report on T2DM, ED (cultural norms of land management and planning, local foodscape, built environment, and neighbourhood deprivation), and their association (involving only risk estimators) will be included. Then, study quality and risk of bias will be conducted according to the combined criteria and ratings from the ROBINS-E (Risk of Bias in Non-randomised Studies—of Exposures) tools and the ‘Effective Public Health Practice Project’ (EPHPP). Finally, the analytical synthesis will be produced using the ‘Synthesis Without Meta-analysis’ (SWiM) guidelines.

**Discussion:**

This systematic review will summarise available evidence on ED associated with T2DM. The results will contribute to improving current knowledge and developing more efficient cross-sectoral interventions in land management and public health in this field of research.

**Systematic review registration:**

PROSPERO CRD42023392073.

**Supplementary Information:**

The online version contains supplementary material available at 10.1186/s13643-024-02488-2.

## Background

Approximately 312 million cases of type 2 diabetes (T2DM) were reported worldwide between 2000 and 2019 [1]. Projections to 2045 are estimated to be approximately 17 million additional cases [1]. T2DM is a complex chronic metabolic disorder [2, 3] mainly characterised by chronic hyperglycaemia [2, 4, 5]. It is caused by a relative insulin deficiency and insulin resistance [4, 5]. Relative insulin deficiency is commonly observed in adulthood [6]. Insulin resistance can often be observed 15 years before relative insulin deficiency [6]. Insulin administration allows patients to reduce the risk of complications and extend their life expectancy. Only prevention can stop the incidence.

**Fig. 1:**
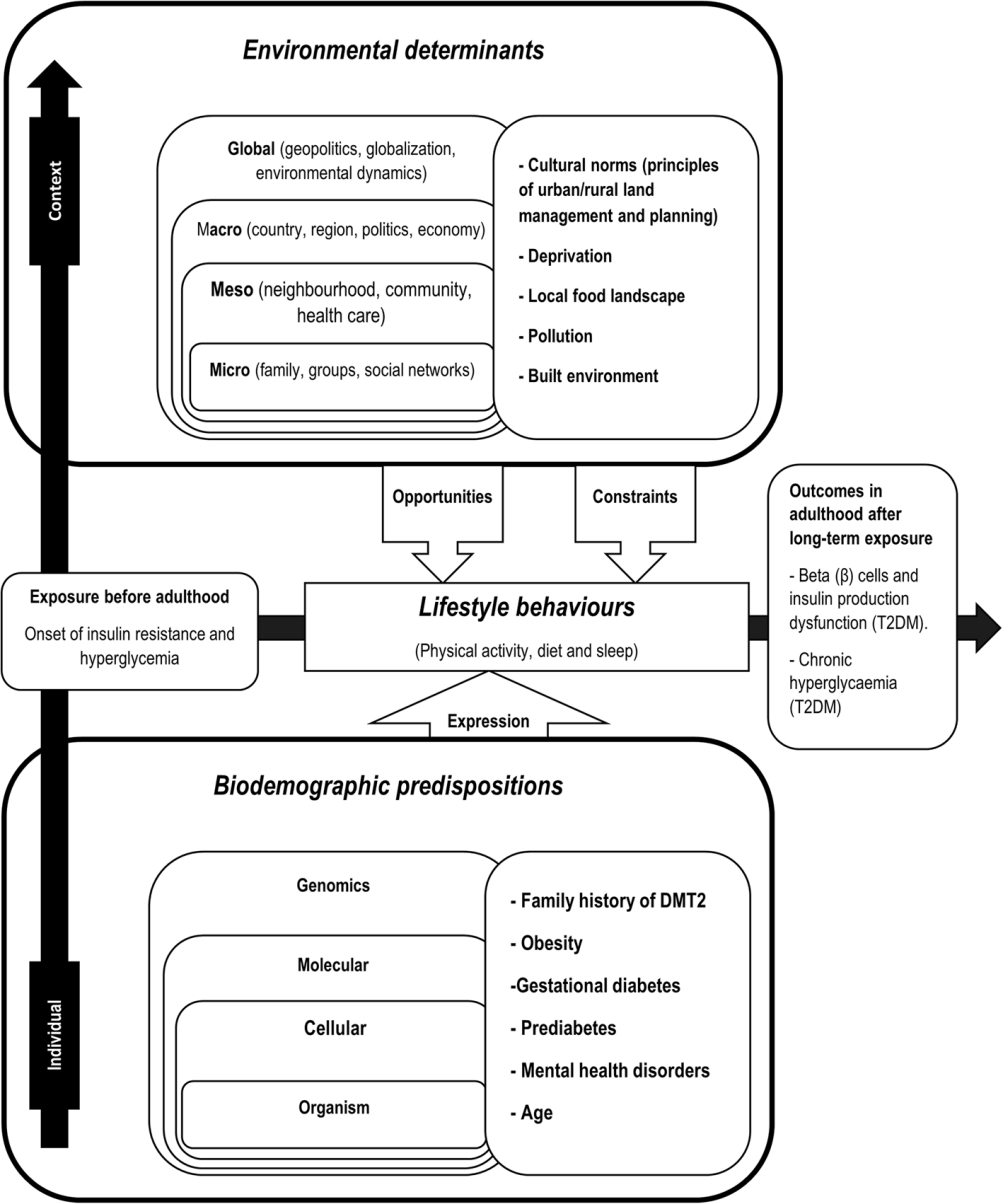
Spatiotemporal and multidimensional socioecological conceptual model for explaining type 2 diabetes mellitus (T2DM). Source of Figure 1: Adapted from A. Lebel [24], inspired by Glass and Mc Atee [25]

The explanatory hypotheses of T2DM, generally put forward, point to the increasingly frequent adoption of unhealthy lifestyle behaviours (a sedentary lifestyle, the abandonment of a balanced diet and a lack of sleep) [4–9]. This is why promoting healthy lifestyle behaviours in the general population and self-management education in at-risk subjects have remained the primary strategy for preventing T2DM. However, the results of this strategy need to be revised [1]. Research [10–16] has shown that adopting healthy lifestyle behaviours depends primarily on an environment that fosters motivation and ensures equitable access to healthy behaviour lifestyle choices. Indeed, a growing body of complementary research recognises that the causes of T2DM are complex (Fig. 1). These causes involve, beyond individual characteristics (biodemographic predispositions [4, 5, 7, 17–21] and lifestyle behaviours [4–8]), contextual characteristics or environmental determinants (ED). These ED are essential to adopting healthy lifestyle behaviours [14, 22, 23].

In the literature, the ED generally mentioned are the local food landscape (food desert) [26–29], the built environment (noise or chemical pollution, non-active/active mobility networks) [27, 30–43], cultural norms of land management and planning (zoning; regional or urban or rural areas land use) [44–46] and material and social deprivation [27, 47, 48] (Fig. 1).

There is evidence from the local food landscape studies that a relatively short distance (compared to fresh food outlets) between fast food outlets and facilities (such as health care, housing, work, education or training) influences food choices [26, 34]. In urban New Zealand, for example, it was found that areas with greater accessibility to fast food outlets were slightly more likely to have a higher risk of T2DM, while areas with greater accessibility to dairy and fruit/vegetable shops had a lower risk [28]. In Quebec, it was found [26] that the risk of consuming unhealthy food at lunchtime is 50% higher among students with access to two or more fast food restaurants within 750 m of their school compared to students without fast food restaurants around their school (odds ratio, 1.5; 95% confidence interval, 1.28–1.75).

About the built environment, studies have shown that, in urban areas, long-term exposure to the neighbourhood that emits or promotes environmental negative externalities, such as unhealthy lifestyle behaviours choices in mobility, increases the risk of developing T2DM. In the case of active transport networks, it has been observed that where distances between the active mobility network and residential locations are relatively large, active mobility and physical activity are less common [34]. In Australia, for example, people who reported that there were no active mobility facilities in the neighbourhood were more likely to develop T2DM [35]. Regarding long-term exposure to noise and chemical pollution, a growing body of evidence argues that emission or promotion of negative environmental externalities such as noise [39, 41] and chemical [37, 38, 40–43] pollution in the neighbourhood of the areas where people spend their most daily time, without regulatory intervention, shapes unhealthy lifestyle behaviours (diet, physical activity and sleep) in long-term residents and increases the risk of developing a T2DM during their life course.

About amenities, evidence supports that long-term exposure to environmental amenities, such as sports facilities, influences the risk of developing T2DM. This evidence concluded that even in populations genetically predisposed to T2DM, the prevalence is mainly determined by ED, as they shape lifestyle behaviours choices [27, 36, 49]. For example, it has been observed that, compared to residential areas within 265 m of a sports-related green space, there was a 9% increase in the prevalence of T2DM in residential areas furthest from such green spaces [36].

Neighbourhood deprivation (material or social) increases the risk of long-term exposure to lifestyle behaviours at risk of T2DM, specifically among people who are experiencing individual deprivation (material or social) [50, 51]. In Saskatchewan (a province in Canada), using the deprivation index for the period 2007–2012, a study [50] showed that, compared to people in the most deprived quintile, those in the least deprived quintile had a lower probability of developing diabetes mellitus (OR = 0.40; 95% CI = 0.18–0.88).

This new knowledge on the relationship between T2DM and ED is helping to stimulate the development of primary prevention policies based on the regulation or legislation (in land use planning and regional development) of environmental changes that impact the choice of healthy lifestyle behaviours associated with diet, physical activity or sleep [52, 53]. However, there are still gaps in current knowledge regarding the following aspects: First, the indicators of ED vary significantly between studies [27, 32, 54]; in addition, studies present results that can be very different and sometimes contradictory, depending on the populations and the location studied [32, 54]; finally, there is currently no up-to-date synthesis of knowledge on the observed impacts of ED and the risk of developing T2DM. These challenges limit the development of public health and spatial planning preventive interventions. A critical analysis of reliable evidence could improve current knowledge and develop more efficient cross-sectoral interventions in land-use planning, regional development, and public health. Previous systematic reviews have addressed this problem with similar approaches [27, 32, 39, 54]. This systematic review aims to improve knowledge about the relationship between the risk of developing T2DM in adulthood and long-term exposure to its ED during childhood or teenage years.

### Research question

Is there evidence to suggest that long-term exposure to ED during childhood or teenage years contributes to increases in the risk of developing a T2DM in adulthood, particularly in urban areas compared to rural areas?

## Methods

The research approach is based on a systematic review methodology of association in exposure [55–57]. It is presented according to the Preferred Reporting Items for Systematic Review and Meta-Analysis Protocols tools (PRISMA-P) [58, 59]. Three information specialists from Laval University libraries were consulted for the development of the search strategy. The selection will follow the ‘population, exposure, comparator, outcome, and study designs’ (PECOS) approach [55, 60]. Quality assessment will be carried out according to the combined criteria and ratings from the ROBINS-E tools (Risk of Bias in Non-randomised Studies – of Exposures) and the ‘Effective Public Health Practice Project’ (EPHPP).

The systematic review will be organised into five main stages. The first stage will involve collecting bibliographical references (data collection), selecting and extracting data on the relationship between ED (exposure) and the risk of developing T2DM (outcome) using eligibility criteria and a search strategy. The second step will be to assess the potential biases and reliability of the selected studies. In the third stage, an analytical synthesis of the evidence will be carried out using the Synthesis Without Meta-analysis (SWiM) guidelines [61]. In the fourth stage, a discussion will be produced. Finally, the main limitations will be highlighted.

This systematic review protocol has been prospectively registered on PROSPERO (https://www.crd.york.ac.uk/prospero): registration number CRD42023392073.

### Eligibility criteria

The inclusion/exclusion criteria (see Table 1), of data collection, will be formulated following the ‘Population, exposure, comparator, outcome, and study designs’ approach or PECOS [55, 60].

**Table 1:** Inclusion/exclusion criteria

Evidence characteristics/Level of triage	Inclusion	Exclusion	Justification
**At level 1 (**first screening or t*itle and abstract screening*)*/***level 2 **(selection based on full text or *full-text* screening)			
**Language**	The study is published in English or possibly in French	The study is published in a language other than English or French	Data collection will be extended to French language publications to contribute to address possible publication bias. But this systematic review project does not have the resources to translate into languages other than English or French
**Year of publication**	The study is published before 2024	The study is published after 31 December 2023	An increasing body of research recognises the involvement of environmental determinants in the risk of developing type 2 diabetes (T2D). This situation illustrates the global awareness of the need to promote neighbourhoods conducive to healthy living behaviours to achieve complete well-being
**Sources of evidence**	- The study was published in a peer-reviewed journal - The study was published in one of the grey literature sources of the Laval University Libraries	Public interest reports or magazines or popular magazines are to be excluded	Although peer review is an essential safeguard of the scientific process, in this research, it is recognised that grey literature sources are a means of addressing potential publication bias
**Study design**	The study may be experimental or non-experimental (cross-sectional, cohorts/longitudinal, case–control) or quasi-experimental (cohorts/longitudinal, case–control), with the aim to explain and quantify the relationship between T2DM (Outcome) and environmental determinants (exposure) using one risk estimator such as Risk ratio (RR), hazard ratio (HR) or odds ratio (OR)	The study uses a qualitative or mixed observational survey methodology	Qualitative data do not quantify the contribution of environmental determinants or objectively compare studies with each other
**Exposure(s)**	The study analyses the correlation, contribution or influence or impact of exposure to local food landscape or built environment or deprivation or rural or urban cultural norms of organization and use of space	The study does not address the contribution, influence, or impact of exposure to environmental determinants	This is an indispensable aspect of answering the research question
**Comparator(s)**	Evidence from control groups made up of individuals who may or may not be predisposed to the risk of T2DM or who are not permanently exposed to an unhealthy environment during their life course (men versus women, urban population vs rural population, unhealthy lifestyle behaviours and obese vs normal weight individuals) will be taken into account	Absence of comparator(s)	Subgroups may be used to improve knowledge of the nature of the relationship observed in this systematic review
**Main outcome(s)**	The study seeks to explain the risk of type 2 diabetes mellitus (causal or correlational)	The study does not seek to explain the risk of type 2 diabetes mellitus	This is an indispensable aspect of answering the research question. Risk was chosen for two reasons: firstly, this systematic review is more concerned with explanatory analyses; secondly, according to exploratory research, it is the most widely used statistical measure of association in studies in this field
**Participants**	Diabetic and nondiabetic individuals 18 years of age or older	Individuals under the age of 18 are included in the study population	Dysfunction in insulin production often appears in adulthood. It is therefore recognized that for environmental characteristics to have an impact on lifestyle behaviors, one must have been exposed to them in the life course at least until adulthood
**Measure(s) of intervention, exposure**	At least one indicator of rural or urban perception of organization and use of space or local food landscape or built environment or deprivation is being defined	There is no definition of environmental indicators	This is an indispensable aspect of answering the research question
**Measure(s) outcome**	At least one indicator of type 2 diabetes (prevalence, incidence for descriptive analyses and risk ratio (RR), hazard ratio (HR), or odds ratio (OR) for explanatory analyses) is being defined (using fasting blood glucose level or FBG and 2-h plasma glucose level or 2hPG level; glycated haemoglobin level or HbA1c; insulin and homoeostatic model assessment of insulin resistance level or HOMA-IR or simply based on administrative health data (for example, the codes E110 to E119 in the 10th revision of International Statistical Classification of Diseases and Related Health Problems or ICD-10) or self-reported cases validated by a concordance study published)	The studied measure of association between DMT2 and the environment is not a risk estimator	Type 2 diabetics account for 90–95% of *Mellitus* diabetes cases worldwide. This is an indispensable aspect of answering the research question. Risk was chosen for two reasons: First, the systematic review is more concerned with explanatory analyses of DMT2; second, based on exploratory research, it is the most widely used statistical measure of association in studies in this field of research

### Population

This systematic review will include all studies with participants aged 18 and over, as it has emerged that it is generally in this age group that dysfunction in insulin production often occurs in cases of type 2 diabetes (DMT2).

### Exposure

Evidence based on social-ecological models has shown that, in urban areas, more than in rural areas during childhood and teenage years, long-term exposure to neighbourhood material deprivation [27, 47, 48], unhealthy built environment [27, 30–33, 44–46] and local foodscape [26–29] contribute to increases the risk of developing a T2DM shape in adulthood, in the form of constraints on choice of healthy lifestyle behaviours. To be included, the evidence sought must have presented the following: firstly, a precise definition of the exposure studied (e.g. local food landscape; noise pollution; chemical pollution; non-active/active mobility networks; amenities; material or social deprivation; zoning; regional or urban or rural areas land use) and secondly, at least one exposure measure.

### Comparators

Evidence from control groups made up of individuals who may or may not be predisposed to the risk of T2DM or who are not permanently exposed to an unhealthy environment during their life-course will be considered. Indeed, subgroups (men versus women, urban population versus rural population, unhealthy lifestyle behaviours and obese versus normal weight individuals) may be used to improve knowledge of the nature of the relationship observed in this systematic review.

### Outcome

Articles that do not present measure of prevalence or incidence of T2DM based on medical screening of T2DM such as fasting plasma glucose (FPG) or glycosylated haemoglobin (A1C) tests, or oral glucose tolerance test (OGTT) coupled with the 2-h plasma glucose test (2hPG), or homoeostatic model assessment of insulin resistance level (HOMA-IR) or equivalent such as administrative health data (e.g. the codes E110 to E119 in the 10th revision of International Statistical Classification of Diseases and Related Health Problems or ICD-10) or self-reported cases validated by a concordance study published will all be excluded.

### Study design

This systematic review will include (see justification in Table 1), to the extent possible, all studies published in English or French until December 31, 2023 (the ‘year of publication’ of the evidence must fall before 2024), including in the grey literature and peer-reviewed scientific journals. Data collection will be extended to French-language publications to contribute to addressing possible publication bias. However, this systematic review project only has the resources to translate into languages other than English or French. December 31, 2023, serves as a pragmatic cut-off date for including recent research without excessively prolonging the review process. This date was selected based on several events that have raised global awareness of the need to promote neighbourhoods conducive to healthy lifestyle behaviours to achieve a state of total well-being. These include the declaration of the Ottawa Charter from the First World Conference on Health Promotion in 1986, the creation in 2005 of the World Health Organization (WHO) Commission on Social Determinants of Health, the publication in 2009 of the report of the Commission on Social Determinants of Health, the eighth World Conference on Health Promotion in Helsinki in 2013, the ninth World Conference on Health Promotion in Shanghai in 2016 and the increasing body of research that recognises the involvement of ED in the risk of developing type 2 diabetes (T2DM). The article’s acceptance year will be considered if it differs from the year of publication.

In addition, the design of the study may be experimental or non-experimental (cross-sectional, cohorts/longitudinal, case–control) or quasi-experimental (cohorts/longitudinal, case–control), with the aim of quantifying the relationship between at least one measure of T2DM frequency (prevalence or incidence) and at least one measure of a dimension of the environment (food desert or local food landscape; noise pollution; chemical pollution; non-active/active mobility networks or amenities; material or social deprivation; cultural norms of land management and planning).

Finally, the measure of association should be a risk estimator such as risk ratio (RR), hazard ratio (HR) or odds ratio (OR).

### Information sources

Two information specialists from Laval University libraries were consulted to identify suitable electronic scientific reference databases. Electronic databases of peer-reviewed scientific journals such as PubMed, Embase, CINAHL, Web of Science, EBSCO and the electronic databases of grey literature of the Laval University Library will be used for data collection.

### Search strategy

Three information specialists from Laval University libraries were also consulted to produce a search strategy. A conceptual design and search equations (queries) (see, e.g. Web of Science Table 2) will be used to identify the studies eligible for selection. The search indexes (keywords or MeSH Terms, subject, topic, title and abstract) will be adapted to each database.

**Table 2:** Keywords used to search for evidence in Web of science

#	Search queries (– Advanced Search Query Builder; Publication date: until December 31, 2023) *?: truncation symbols for easy search; AND, OR’ …’: main search operators
**1**	TI = (‘*ype 2 diabetes’ OR ‘*iabetes *ellitus’ OR ‘*on-insulin-dependent’ OR ‘*on-insulin-dependent diabetes’ OR ‘*iabète de type 2’ OR diabetes)
**2**	((**#1** AND AB = (‘environment* risk factors’ OR ‘geographic* variation’ OR ‘geographic* distribution’ OR ‘geographic* inequit*’ OR ‘spatial disparit*’ OR ‘geographic*’ OR ‘communit* type*’ OR ‘physical* work environment*’ OR ‘environment* factor*’ OR ‘environmental condition*’ OR ‘neighbo? hood environment*’ OR ‘neighbo? hood physical*’ OR ‘neighbo? hood qualit*’ OR ‘built environment*’ OR ‘environnement* social’ OR ‘neighb? hood road environment*’ OR neighbo? hood OR environment* OR ‘geographic* area’ OR ‘perceived environment’ OR ‘neighbo? hood walk*’ OR ‘neighbo? hood built environment*’ OR geo* OR spati*)
**3**	((**#1** AND AB = (‘food environment*’ OR ‘food desert*’ OR foodscape* OR ‘food access’ OR ‘eat* place*’ OR ‘food store*’ OR ‘food suppl*’ OR ‘food establishment*’ OR ‘grocery store*’ OR dair* OR hunger OR ‘access to health option*’ OR ‘access to food*’ OR ‘health* food environment*’ OR greengrocer* OR ‘food insecurit*’ OR ‘food avaibilit*’ OR ‘fast-food outlet*’ OR ‘fast food restaurant*’ OR ‘retail food environment*’ OR ‘convenience store*’ OR ‘fruit* and vegetable*’ OR market* OR ‘food that support health*’ OR ‘eating pattern*’ OR supermarket*)
**4**	((**#1** AND AB = (‘air & water qualit*’ OR ‘public transport*’ OR ‘active transport’ OR ‘transportation’ OR ‘green space*’ OR green* OR park* OR ‘recreational facilit*’ OR amenit* OR ‘health* service*’ OR ‘access to health* care’ OR ‘access to primary care*’ OR ‘access to exercise*’ OR ‘health literac*’ OR ‘health* coverage’ OR housing OR playground* OR urban OR ‘urban area*’ OR ‘rural area*’ OR rural OR ‘public transit station*’ OR ‘open space*’ OR ‘recreatio* walk*’ OR ‘leisure walk*’ OR walk* OR ‘nonmotorized transportation’)
**5**	(**#1** AND AB = (‘street connectivit*’ OR ‘road traffic*’ OR walkabilit* OR sidewalk OR ‘land-use mix*’ OR ‘manhattan distance’ OR ‘shortest network time’ OR ‘shortest network distance’ OR ‘euclidean distance’ OR mixit* OR ‘road environment*’ OR ‘engineering of road environment*’ OR ‘street network’ OR ‘pedestrian network’)
**6**	((**#1** AND AB = (‘housing instabilit*’ OR ‘labor hous*’ OR ‘qualit* of hous*’ OR ‘household socioeconomic* level’ OR ‘qualit* of care*’ OR ‘socioeconomic* deprivation’ OR ‘material deprivation’ OR ‘macroeconomic* polic*’ OR income OR occupation OR ‘social deprivation’ OR ‘community safet*’ OR safet* OR ‘social securit* insurance’ OR ‘social cohesion’ OR ‘population densit*’ OR vandalism OR ‘social intégration’ OR ‘famil* and social support’ OR ‘support system*’ OR ‘communit* engagement’ OR ‘civi* participat*’ OR ‘ethnicit*’ OR racism OR ‘social class’ OR gender OR descrimination OR ‘crime and violence’ OR employment OR ‘income tax*’ OR debt* OR expens* OR ‘medical bill*’ OR poverty OR ‘enrollment in higher education’ OR education* OR ‘college degree’ OR ‘higher education’ OR ‘vocational training*’ OR ‘higher school graduation’ OR literac* OR ‘language and literac*’ OR ‘early childhood education & development’ OR ‘social protection’ OR ‘culture & societal value*’ OR governance OR ‘social environment*’)
**7**	**#2** OR **#3** OR **#4** OR **#5** OR **#6**

### Data management

The bibliographic references found in the above-mentioned electronic databases of grey literature and peer-reviewed scientific journals will be exported and assembled in a single directory to facilitate automatic processing. The ‘Covidence’ application will be used to store them and download the full texts.

### Selection process

**Fig. 2:**
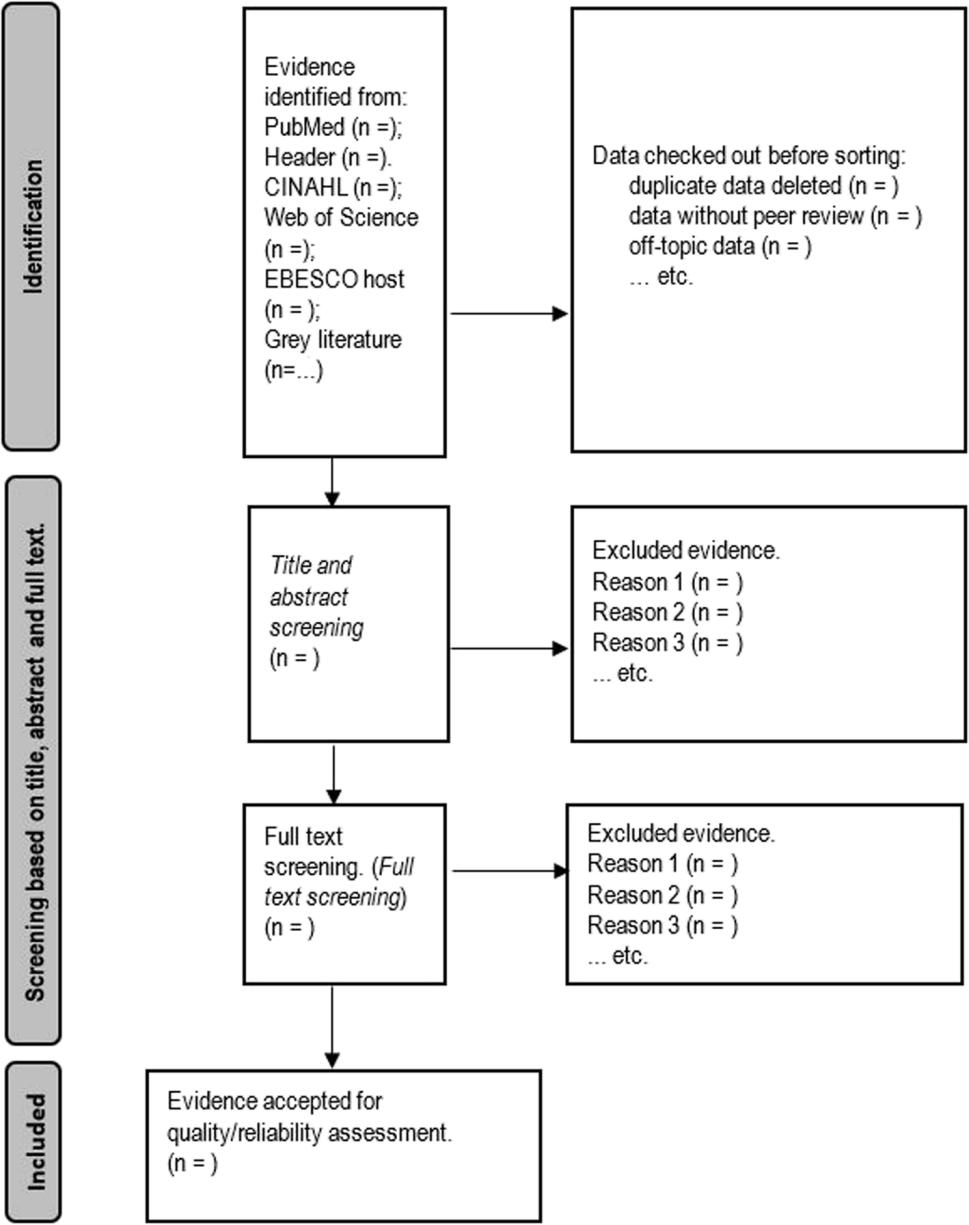
Expected evidence collection flow diagram adapted from PRISMA-STATEMENT

Two reviewers will independently perform the article screenings in the ‘Covidence’ application using the inclusion/exclusion criteria mentioned above. A third reviewer will intervene mainly in case of selection conflicts. The selection will be made at two levels. Title and abstract screening will be performed at the first level and full-text screening at the second level (see the expected flow chart in Fig. 2).

### Data extraction process

One reviewer will perform data extraction in the ‘Covidence’ application using a data extraction table validated consensually by all the reviewers. The choice of the main characteristics to be extracted will be in line with the guidance provided by tools such as the ‘Effective Public Health Practice Project’ (EPHPP) or ‘Risk of Bias in Non-randomised Studies – of Exposures’ (ROBINS-E), the ‘Cochrane Handbook for Systematic Reviews of Interventions’ [62] and ‘The Joanna Briggs Institute’ [56] approach (see, e.g. in Table 3 below). These primary characteristics are names of authors, year of publication, journal name, study design, type of study, date of the study, location of the study site, nature of the relationships studied, participation, age of participants, sex of participants, type of exposure, exposure measurements, the exposure outcome, the measurement of exposure outcome, potential comparators or confounding or confounding variables, type of modelling, regression model, association measures, results of the association measure, key findings and relevant comments. The observation of the state of the relationship and the life-course perspective will be drawn from the methodological details, the results of the association measurement, the main conclusions and the authors’ relevant comments.

**Table 3:** Example of a data extraction table

Key elements to extract		Details/clarifications
**Sources**	Authors	Identify the authors of the study
	Year of publication	Identify the year the study was published
	Journal name	Identify the publication review
**Methods**	Research specifications/study design	Identify the type of survey methodology such as experimental or non-experimental (cross-sectional, cohorts/longitudinal, case control) or quasi-experimental (cohorts/longitudinal, case control)
	Type of study	Identify the purpose of the study: etiological/analytical or the search for a relationship between a disease and its alleged factors
	Date of study	Please specify the period during which the study took place
	Location of the study site	Identify the country or environment or environment in which the study is conducted
	Nature of the relationships studied	Specify if the nature of the relationships studied is correlational or causal
**Participants/population**	Participation	Total number of study participants; participation rate
	Age	Mean, median, standard deviation or extent
	Sex	Number or percentage
**Exposition**	Exposure	Dimensions of the exposure studied
	Exposure measures	The indicators corresponding to each dimension of exposure studied
**Effect of exposure**	Outcome	This is the result of the screening type 2 Diabetes Mellitus (T2DM)
	Outcome measures	This is the frequency indicator for T2DM
**Adjustment factors/comparators**	Potential confounding or confounding variables	These are the main factors associated with exposure and exposure outcome, independently
**Modeling**	Type of modeling	Ecological; multilevel or individual/traditional
	Regression model	Statistical regression model (binomial, Poisson, etc.)
	Association measures	Define the risk measure used
**Modelling results**	Results of the association measure	This involves including the number of participants allocated for each intervention dimension and the summary of data for each intervention dimension (a contingency table for dichotomised data or mean and standard deviation for continuous data). Estimates of effect with confidence intervals and *p values* if available should also be included
	Key findings of the study authors	The key findings of the study
	Relevant comments	All comments from the authors deemed relevant for a better understanding of the results of the study

### Risk of bias

Two reviewers will independently perform bias/quality assessment using the ‘Covidence’ application. A third reviewer will intervene mainly in case of bias/quality assessment results selection conflicts.

The evaluation of the risk of bias of the selected evidence will be carried out according to the combined criteria and ratings for non-experimental and quasi-experimental studies from the ‘Effective Public Health Practice Project’ (EPHPP) (see Additional file 2) and the ‘risk of bias in non-randomized studies–of exposures’ (ROBINS-E) tools (see Additional file 4).

There are few tools for analysing the methodological quality of non-experimental and quasi-experimental studies with an aetiological focus based on purely quantitative data and applicable indiscriminately and simultaneously to various methodological profiles. The best known are the ROBINS-E tools, the ‘Newcastle–Ottawa scale’ (NOS) for assessing the quality of non-randomised studies in meta-analyses, and the ‘quality assessment tool for quantitative studies’ from the EPHPP. The ROBINS-E tool and the ‘quality assessment tool for quantitative studies’ propose a rating technique promoted by ‘The Public Health Agency of Canada’ (PHAC) [63] that consists of awarding ‘strong’, ‘moderate’ and ‘weak’ ratings according to the quality of the study. The biases assessed are practically identical or complementary.

Combining the ‘quality assessment tool for quantitative studies’ and ROBINS-E tools consists of two tasks. First, several sub-types of selection bias (e.g. ‘blinding’ or ‘withdrawals and drop-outs’), information bias (e.g. ‘non-differential misclassification’ or ‘differential misclassification’) or confounding bias (e.g. ‘competitive risk bias’ or ‘indication bias’) can have an impact on the quality of a study, particularly non-experimental or quasi-experimental studies. While ‘Quality Assessment Tool for Quantitative Studies’ and ROBINS-E each partially assess these biases, merging their questions into a single tool addressing different types and subtypes of biases overcomes this limitation. In addition, reformulating their information questions (often introduced by words such as ‘who’, ‘what’, ‘where’, ‘when’ or ‘how’) into closed questions (allowing only ‘yes’ or ‘no’ answers) will reduce reporting bias and improve repeatability and reproducibility.

## Criteria and ratings for assessing the reliability of evidence

Criteria for the reliability of the evidence will be based on the standards of the EPHPP and ROBINS-E tools. This evaluation will consider topics such as the risk of bias in the selection of study participants, the risk of bias due to post-exposure interventions, the risk of bias due to confounding, the risk of bias related to exposure measurement, the risk of bias due to missing data and the risk of bias in the selection of reported results. The reliability will depend on the result of the evaluation of the quality of the studies analysed (see examples in Table 4).

**Table 4:** Quality assessment criteria for selected studies

Evaluation questions (1 = yes; 2 = partially; 3 = no; 4 = not stated; 5 = not applicable)														Evaluation results	
Author, year, reference	A) Selection bias				Information bias				Confounding					**Total # Yes**	**Overall quality: strong, 10 yes and + ; moderate, 6–9 yes; weak, 1–5 yes**
	1) Did 80–100% of selected individuals agree to participate?	2) Were all participants selected or recruited from the same population, subject or unit of study, or similar populations, including the same period?	3) Selection of participants into the study, or into the analysis, not based on participant characteristics observed after the start of the exposure or exposure window being studied	4) Does 80–100% of participants completed the study?	5) About the risk of bias arising from the measurement of outcomes, measurement or ascertainment of the outcome has not differed between exposure groups or levels of exposure	6) Does the measured exposure (including changes over time) well-characterize the exposure metric specified to be of interest in this study?	7) Were exposure have been assessed more than once over time of observation?	8) Assessment of the outcome has not been influenced by knowledge of participants’ exposure history?	9) Have the main potential confounders been measured?	10) Is the percentage of confounding factors controlled at least equal to 60–79%?	11) Were confounding factors that were controlled for, and for which control was necessary, measured validly and reliably by the variables available in this study?	12) Have the main potential confounders or confounders been statistically adjusted for their impact on the relationship between exposure and outcomes?	13) Did the authors use an analysis method that was appropriate to control for time-varying confounding?		

Source: Based on the Risk Of Bias In Non-randomized Studies – of Exposures (ROBINS-E) assessment tool (see Additional file 4) and the Effective *Public Health Practice Project* (EPHPP) (https://www.ephpp.ca/quality-assessment-tool-for-quantitative-studies/ or https://merst.healthsci.mcmaster.ca/ephpp/) (see Additional file 2) [64, 65]

The global rating of the reliability for one scientific article included in this review is attributed as follows:

- Strong (1) if the study records a number of 35 or more ‘yes’ responses
- Moderate (2) if the study registers between 21 and 34 ‘yes’ responses
- Weak (3) if the study registers fewer than 21 ‘yes’ responses

### Analytical synthesis

This step will be structured around nine items, in line with the ‘synthesis without meta-analysis’ (SWiM) guidelines [61].

First, the studies will be grouped according to the geographical region of origin of the study (e.g. North America, South America, Eastern Europe, Western Europe), the individual characteristics of the participants (sex and age group), exposition (exposure and exposure measures), the effect of exposition (outcome and outcome measures), modelling (type of modelling, statistical regression model, standardised metric of association measures) and study design (experimental or non-experimental (cross-sectional, cohorts/longitudinal, case–control) or quasi-experimental (cohorts/longitudinal, case–control). Similarities and dissimilarities will be identified and highlighted in the descriptions of these groups.

Second, the description of the outcome (the screening result of T2DM, such as FBG or HbA1c, and the frequency indicator for T2DM, such as prevalence or incidence) and standardised metric of association measures (e.g. RR, HR, OR), as reported in the studies, will be produced.

Third, the ‘statistical synthesis methods when a meta-analysis of effect estimates is impossible’ will be used for the synthesis methods point. These include ‘summarising effect estimates’ or ‘combining *P* values’ [66]. This choice is due to the incomplete data resulting from the diversity of methods and results in this field of research.

Besides, the risk of bias assessment (only studies with ‘strong’ and ‘moderate’ quality), the study design (cohorts or longitudinal) and the exposure effect (a risk estimator such as RR, HR or OR based on T2DM incidence) will be the main criteria used to prioritise results for summary and synthesis.

Next, the investigation of heterogeneity in reported effects will consist of classifying ordering tables or structuring figures by geographical region of origin of the study, the individual characteristics of the participants, exposure, outcome and type of modelling (ecological, multilevel or individual/traditional). The heterogeneities highlighted will involve capitalising on the approach that can reduce potential methodological biases as far as possible and identify the primary research needs.

In addition, the assessment of certainty will be based on the ‘Grading of Recommendations, Assessment, Development and Evaluations’ (GRADE) approach [67]. Where the data allow, the characteristics of the studies will be taken into account, such as the precision of the result (confidence interval), the number of studies and participants, the consistency of the effects between the studies, the risk of bias in the studies, the consistency between the research question and the results of the studies and the risk of publication bias, in order to determine the level (‘high’, ‘moderate’, ‘low’, ‘very low’) of certainty of the synthesis of the results.

Equally important, a table alphabetically ordering studies by study ID will be created using Microsoft Excel. Box-and-whisker plots of risk estimators (such as RR, HR or OR) for all outcomes and separately by the global rating of the reliability or other studies characteristics will be created using Microsoft Excel.

Then, the method used to describe the various results (investigation of heterogeneity and synthesis findings) will consist of comparing them with the research question, the method of synthesis used (‘summarising effect estimates’ or ‘combining *P* values’), the characteristics of the studies, the effect of the exposure studied and its confidence interval.

Finally, it should be noted that the main limitation of statistical synthesis methods when a meta-analysis of effect estimates is not possible (‘summarising effect estimates’ or ‘combining *P*-values’) is that they limit informed decision-making. However, they allow for improving the transparency and reproducibility of analyses and identifying the primary research needs.

Based on this analysis, conclusions will be drawn about the relationship between environmental conditions and T2DM from life-course perspective, noting the contexts in which the studies were carried out and the limitations involved.

## Discussion

The interpretation of the results of the systematic review will be discussed in this section. It will be based on the results of the analytical and narrative synthesis. Thus, all results that met all conditions up to reliability will be included.

In the first, the general level of reliability of the data will be discussed. Indications will also be given on the specific reliability of the data on which the conclusions are based.

In addition, the following points will be developed: (i) A summary of the main results will be produced; (ii) the general interpretation of the results of the research question will be carried out; (iii) the contribution of the research results of this systematic review of what exists will be highlighted; (iv) the strengths and limitations of the scope of the systematic review will be discussed; and (v) the methodological gaps that remain in the analysis of the relationship between ED and T2DM will also be presented. Emphasis may be placed on the impact of these gaps in knowledge in this field of research. Beyond the research advances, the results could help to guide cross-sectoral policies and strengthen informed decision support for policy-makers in land-use planning, regional development and public health, for better targeting and coordination of T2DM prevention.

## Limitations

The main limitation of this protocol remains a relatively high number of results that the search strategy will produce, depending on the electronic databases used. An initial search was carried out to ensure that the keywords for the main concepts matched the evidence found. Results from peer-reviewed scientific journals (PubMed, Embase, CINAHL, Web of Science, EBSCO) varied around 1500, while those from grey literature sources varied around 4. This is because the keyword ‘diabetes’, which produces more results than the keywords ‘type 2 diabetes’ or ‘diabetes mellitus’ or ‘type 2 diabetes mellitus’, has been added to the search strategy. It became apparent during the exploration of the electronic databases that many authors prefer to use the keyword ‘diabetes’. The fact that T2DM accounts for around 90% of cases of DM worldwide can probably help explain this vocabulary choice [68]. More time will be allocated to the title and abstract screening stage to address this limitation.

In addition, meta-analyses will not be included in this research. A meta-analysis, as a complementary study to this systematic review, is planned for publication later. The methodological approach, the acquisition of human resources (e.g. recruitment of meta-analysts) and financial resources (e.g. funding) is currently being considered for this purpose.

Finally, due to the above logistical constraints, scientific studies published in languages other than English and French may not be used.

### Supplementary Information

**Additional file 1. **PRISMA-P checklist of crucial aspects of a protocol paper.

**Additional file 2. **A quality assessment tool for quantitative studies of the EPHPP.

**Additional file 3.** Consent for publication_ In French.

**Additional file 4. **ROBINS-E_template.

## Data Availability

Not applicable.

## Abbreviations

ED: Environmental determinants
PRISMA-P: Preferred Reporting Items for Systematic review and Meta-Analysis Protocols
PECOS: Population, exposure, comparator, outcome, and study designs
PHAC: Public Health Agency of Canada
EPHPP: Effective Public Health Practice Project
T2DM: Type 2 diabetes mellitus
GRADE: Grading of Recommendations, Assessment, Development and Evaluations
SWiM: Synthesis without meta-analysis
FPG: Fasting plasma glucose
FBG: Fasting blood glucose level
HbA1c: Glycated haemoglobin level
A1C: Glycosylated haemoglobin tests
OGTT: Oral glucose tolerance test
2hPG: 2-Hour plasma glucose test
HOMA-IR: Homoeostatic model assessment of insulin resistance level
RR: Risk ratio
HR: Hazard ratio
OR: Odds ratio
