# Supplementary material for: Contribution of environmental determinants to the risk of developing type 2 diabetes mellitus in a life-course perspective: a systematic review protocol
Source: Syst Rev. 2024 Mar 1;13:80. doi: 10.1186/s13643-024-02488-2 (PMC10908215; doi:10.1186/s13643-024-02488-2)
Supplement: Supplementary file 3 — Additional file 3. Consent for publication_ In French. [file 13643_2024_2488_MOESM3_ESM.pdf]

UNIVERSITÉ LAVAL

**Faculté d'aménagement, d'architecture, d'art et de design**
École supérieure d'aménagement du territoire et de développement régional

Québec, le 24 novembre 2022

À : *Yannick Mengue, candidat à la maîtrise en ATDR, ÉSAD, Université Laval*

De : *Alexandre Lebel Ph.D., directeur de recherche, ÉSAD, Université Laval*

CC : *Jean Dubé Ph.D., codirecteur de recherche, ÉSAD, Université Laval*
*Geneviève Cloutier, Ph.D., examinatrice, ÉSAD, Université Laval*
*Claude Lavoie Ph.D, Directeur de l'ÉSAD, Université Laval*

Objet : Évaluation de la présentation du projet de maîtrise (AME-6012)

Monsieur Yannick Mengue,

Suivant la présentation de votre projet de maîtrise qui a eu lieu le mardi 22 novembre 2022, les membres du comité d'évaluation ont de votre proposition pour la réalisation d'une maîtrise avec mémoire à l'École supérieure d'aménagement du territoire et de développement régional (ÉSAD).

J'ai le plaisir de vous confirmer que vous avez réussi l'exercice à la satisfaction de tous les membres du comité. Ceux-ci jugent que vous avez toutes les qualifications requises pour la poursuite de votre projet de recherche à l'ÉSAD, et ils vous accordent la **note B+**.

Les membres du comité soulignent les qualités de votre travail, et portent à votre attention certains éléments à considérer dans la perspective de bonifier vos activités de recherche à venir:

- *le candidat a offert une prestation qui a atteint les attentes à plusieurs égards, incluant la qualité du document écrit, et une bonne maîtrise de la méthode proposée;*
- *le document écrit était bien structuré, mais il aurait bénéficié d'une meilleure assise sur un cadre conceptuel spécifique, ainsi que des précisions sur les critères de sélection et d'exclusion de la revue de littérature systématique;*
- *de la même manière, la présentation orale était trop courte et aurait bénéficié des mêmes détails théoriques et méthodologiques que le document écrit;*
- *les réponses aux questions étaient généralement correctes, mais souvent hésitantes et parfois minimalistes;*
- *le cadre analytique est cohérent, mais mériterait lui aussi des précisions sur les déterminants environnementaux, particulièrement en ce qui concerne la formulation de la question de recherche et des hypothèses;*
- *le candidat peut avoir confiance en ses capacités à réaliser le projet de recherche proposé, les membres du comté d'évaluation se sont également exprimés favorablement à cet égard.*

Veuillez recevoir toutes mes félicitations, et je vous souhaite bon succès dans la réalisation de votre maîtrise avec mémoire à l'ÉSAD.

**Alexandre Lebel**
Directeur de recherche
